# Completion rate of small bowel capsule endoscopy is higher after erythromycin compared to domperidone

**DOI:** 10.1186/1471-230X-14-162

**Published:** 2014-09-19

**Authors:** Jessie Westerhof, Rinse K Weersma, Reinier A Hoedemaker, Jan J Koornstra

**Affiliations:** Department of Gastroenterology and Hepatology, University Medical Centre Groningen, University of Groningen, PO Box 30001, 9700 RB Groningen, The Netherlands

## Abstract

**Background:**

In up to 30 percent of small bowel capsule endoscopy procedures, the capsule does not reach the cecum within recording time. A prolonged gastric transit time has been recognized as a risk factor for incomplete capsule endoscopy. The aim of this study was to analyze if a single dose of orally administered erythromycin prior to capsule endoscopy results in a higher completion rate compared to orally administered domperidone.

**Methods:**

Single centre, non-concurrent prospective cohort study, 649 capsule endoscopy procedures were included. Cecal completion rates, gastric and small bowel transit times and diagnostic yield were analyzed.

**Results:**

239 patients received erythromycin, 410 patients received domperidone. The cecal completion rate was 86% after erythromycin versus 80% after domperidone (p = 0.03). After excluding known risk factors for incomplete capsule endoscopy such as hospitalization and previous abdominal surgery, erythromycin still resulted in an increased completion rate (p = 0.04). Median gastric transit time was lower after erythromycin compared to domperidone (13 min versus 22 min, p < 0.001). Median small bowel transit times were similar in both groups (236 min versus 248 min, p = 0.21).

**Conclusions:**

In this study, the largest to date on this subject, the cecal completion rate was higher with erythromycin than with domperidone, but there was no difference in the diagnostic yield.

## Background

Small bowel capsule endoscopy (CE) has become an established technique to explore the small bowel. An important limitation of small bowel capsule endoscopy is that in up to 30% of the procedures, visualization of the small bowel is incomplete because the capsule does not reach the cecum within recording time. This may lead to inconclusive procedures and the need for repeated investigations [1].

We have previously shown that one of the risk factors for incomplete CE is a long gastric transit time (GTT) [1]. Hence, there is a rationale to use prokinetic agents prior to the procedure to decrease GTT and thereby potentially increase the rate of complete small bowel examinations. A prokinetic agent that has been studied often in this setting is erythromycin [2–6]. Next to its bactericidal activity, erythromycin induces high amplitude gastric propulsive contractions by activating the gastric phase III interdigestive migrating motor complex, thereby accelerating gastric emptying [7–12].

A recent meta-analysis on prokinetic agents and the completion rate in small-bowel capsule endoscopy showed that the use of prokinetics overall improved the completion rate, without influencing the diagnostic yield [13]. Previous studies on the effects of erythromycin in capsule endoscopy suggested no beneficial effect of erythromycin on capsule endoscopy completion rates [2–6], but it must be realized that these studies are probably underpowered, as no study included more than 50 patients per treatment arm. The primary aim of the our study was to analyze whether a single dose of orally administered erythromycin prior to capsule endoscopy results in higher completion rates compared to domperidone in a prospective cohort study in 649 patients. Secondary endpoints were differences in GGT, small bowel transit time (SBTT) and the diagnostic yield of CE.

## Methods

### Patients

We performed a prospective non-concurrent cohort study. Our study has adhered to the STROBE guidelines for observational studies. Data from all consecutive CE procedures performed at our Department, which is a tertiary referral center, between March 2005 and May 2010, were collected. Between March 2005 and July 2008, patients received 10 mg of oral domperidone directly before the procedure as part of the preparation. From July 2008 to May 2010, we changed our strategy to giving 250 mg of erythromycin orally 1 hour before the procedure. The cohorts were therefore sequential and data were analyzed retrospectively in a so-called non-concurrent cohort study design.

Collected data included patient demographics, hospitalization and previous abdominal surgery, indication for the procedure and CE findings. The most relevant findings obtained from CE were documented and categorized according to standard terminology [14] as angiectasia(s); ulcer(s); bleeding of unknown origin; erosion (s); polyp(s)/tumor(s); incidental abnormality of esophagus, stomach, or colon; no abnormality; or unable to make a diagnosis.

Main outcome measurements were completion rates, GTT, SBTT, and the diagnostic yield. The GTT was defined as the time, in minutes, from the first image of the stomach until the first image of the duodenum. The SBTT was defined as the passage time, in minutes, from the first image of the duodenum until the first image of the cecum. If the capsule did not reach the cecum within recording time, the SBTT was calculated by subtracting the GTT from the total recording time. CE was considered complete when the cecum was reached within recording time. The diagnostic yield was defined as the percentage of positive findings of CE.

### CE procedure

All patients were given standardized instructions before the procedure, and informed consent was obtained. The patients were asked to stop iron supplements seven days before CE and to use a low-fiber diet three days before the procedure. The patients started a fasting period at midnight before the procedure. Bowel preparation consisted of the ingestion of four liters of a polyethylene glycol solution (Colofort^®^), 3 liters the evening before the procedure and 1 liter in the morning. Bowel prepration was given as it has been shown that it increases the diagnostic yield of the procedure [15]. The capsule (PillcamSB; Given Imaging Ltd, Yoqneam, Israel) was swallowed in the morning. The patients were allowed to drink fluids after three hours and to consume a light meal after five hours. Before capsule ingestion, 10 mL of antifoam was given. It must be noted that during the study period no other changes were made with respect to the capsule endoscopy procedure except the choice of prokinetic agent.

From March 2005 to July 2008, 10 mg domperidone was given orally directly prior to the procedure. Data from 410 consecutive patients were collected. Starting July 2008, 250 mg of erythromycin instead of domperidone was administered orally one hour before capsule ingestion. Using this regimen, data from 239 consecutive patients were collected. Patients in whom the capsule was released by an endoscope in the duodenum were excluded. Technical failures were also excluded. CE recordings were reviewed by two gastroenterologists, experienced with capsule endoscopy (RKW and JJK). Controversial findings were discussed, and consensus was reached upon the final diagnosis. During the study period no modifications were made in the capsule technology with 8 hours of recording time during the entire period. The study protocol was approved by the Local Institutional Review Board: the Medical Ethics Committee of the University Hospital Groningen.

### Statistical analysis

Statistical analysis was performed by using the Student *t* test for normally distributed continuous variables and the Chi-square test for non-continuous variables. The Mann–Whitney test was used for data that were not normally distributed. P-values below 0.05 were considered significant. SPSS 16.0 for Windows software (SPSS Inc., Chicago, IL, USA) was used for all statistical analyses.

## Results

### Patients and findings

In total, 649 patients were included. 410 patients received domperidone and 239 patients received erythromycin. Baseline patient characteristics, indications for CE and findings of the procedures are summarized in Table 1. Overall, the two groups were fairly comparable. Median age was slightly lower in the domperidone group: 55 years (range 9–93) and 60 years (range 18–89) in the erythromycin group (p = 0.03) and in the domperidone group, the indications for the procedure was more often occult gastrointestinal bleeding.

**Table 1:** **Baseline patient characteristics, indications and findings**

	Domperidone	Erythromycin	p
Number of patients	410	239	
Male (n,%)	192 (47)	108 (45)	0.69
Age, years (median)	55 (9–93)	60 (18–89)	0.03
Indications (n,%)			0.01
- occult gastro-intestinal bleeding	253 (62)	183 (77)	
- overt gastro-intestinal bleeding	53 (13)	20 (8)	
- suspected Crohn’s disease	104 (25)	36 (15)	
Findings (n,%)			
- angiodysplasia(s)	58 (14)	57 (24)	
- erosion(s)	62 (15)	14 (6)	
- ulcera	25 (6)	11 (5)	
- polyp(s)/tumor(s)	37 (9)	19 (8)	
- active bleeding	22 (5)	5 (2)	
- no abnormalities	206 (50)	133 (56)	

### Number of complete CE procedures

The number of complete CE procedures in the erythromycin group was 86% (95% CI 81-90%) versus 80% in the domperidone group (95% CI 76-83%; p = 0.03). Previous small bowel surgery and hospitalization are known risk factors for incomplete CE procedures [1]. To exclude the influence of these known risk factors we also performed the analysis after exclusion of patients with these know risk factors in both groups. When these patients were excluded, leaving 488 patients for analysis, the difference between the domperidone and the erythromycin group was still significant (91% completion rate in the erythromycin group, 85% in the domperidone group, p = 0.035).

### Gastric and small bowel transit time

**Figure 1:**
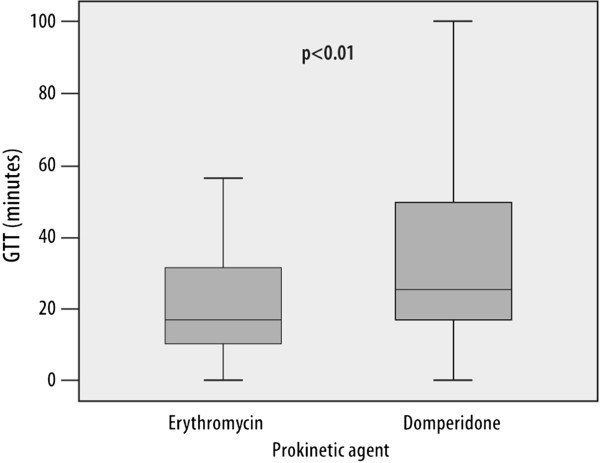
**Box plot depiction of gastric transit times in the erythromycin group compared to the domperidone group (median 13 versus 22 minutes, p < 0.01).**

The GTT was significantly shorter (median 13 minutes, 25 and 75 percentiles; 12 and 54 minutes) in the erythromycin group than in the domperidone group (median 22 minutes, 25 and 75 percentiles; 7 and 27 minutes), p < 0.01 (Figure 1). There was no difference in SBTT between both groups: median 248 minutes in the domperidone group (25 and 75 percentiles: 197 and 336 minutes) and 237 minutes (25 and 75 percentiles; 156 and 347 minutes) in the erythromycin group, p = 0.21.

### Diagnostic yield

There was no significant difference in the diagnostic yield between both groups: 50% in the domperidone group versus 44% in the erythromycin group, p = 0.18.

## Discussion

In this prospective non-concurrent cohort study, the rate of complete capsule endoscopy procedures was higher using the prokinetic agent erythromycin compared to domperidone. We found that erythromycin shortened the gastric transit time without influencing the small bowel transit time or diagnostic yield. These findings remained significant after excluding hospitalized patients and patients with previous small bowel surgery, known risk factors for incomplete CE procedures.

Previous studies found conflicting data on the use of erythromycin as a prokinetic agent in capsule endoscopy. Most studies found an acceleration of the GTT, although not significant in all studies, without actual improving completion rates [2–6, 13]. The latter is undoubtedly due to small patients groups in previous studies. Our power analysis revealed that at least 199 patients needed to be studied in both arms, whereas previous studies included no more than 50 patients in each treatment arm.

In previous studies erythromycin was effective in reducing GTT in humans and animals with gastric paresis [11, 12]. In the literature on erythromycin as a prokinetic agent in CE, some studies found a significantly reduced GTT [3, 4] but none reported an effect on SBTT, total transit time or improved completion rates [2–6, 13]. Some found a non-significant reduction in GTT [2, 5, 6]. Most studies compared erythromycin to placebo or no agent with equal bowel preparations in both groups [2–5]. In one study bowel preparation was different in the study groups making the interpretation of the effect of erythromycin difficult [6]. Finally, one study used real time viewing with on demand use of erythromycin to facilitate the passage of the capsule into the small bowel. In this study the completion rate of CE was higher in the group that used erythromycin on demand [16].

Since we previously showed that a prolonged gastric transit time was a risk factor for incomplete capsule endoscopy procedures [1], and there was evidence that erythromycin may decrease GGT, we started administering erythromycin prior to CE. We used a dosage of 250 mg erythromycin given orally 1 hour before the procedure, because it was safely used in previous studies and reduced GTT in those studies [2, 3, 5]. However, a study on pharmacokinetics of erythromycin as a prokinetic agent showed that erythromycin suspension may be superior to administration of a tablet [17]. It can be speculated that the results might improve with the use of erythromycin suspension.

A strong point of our study is that this is by far the largest study population on this subject, supported by fulfillment of power calculation criteria. A limitation of our study design is that it was non-randomized, and also not placebo-controlled. Instead, we compared erythromycin to domperidone before and after a certain time point. It should be noted that except for the change in prokinetic agent no other modifications were made with respect to capsule preparation or protocol during the entire study period. We initially used domperidone as a prokinetic agent prior to capsule endoscopy, because several studies at that time showed improved gastric emptying in patients with diabetic gastroparesis [18]. A recent study on domperidone showed that it also improves the completion rate of capsule endoscopy without influencing the diagnostic yield [19]. Furthermore, it lacks the extrapyramidal adverse effects of the more widely used metoclopramide. From July 2008 onward, erythromycin was given instead of domperidone. Our results may have been different when the drug had been compared to placebo. It must be noted that the mean gastric transit time in our study was 13 minutes for erythromycin and 22 minutes for domperidone, compared to 34 minutes in a recent large study on capsule endoscopy in 540 outpatient CE procedures receiving no prokinetic agent [20]. In inpatient procedures, it was even longer, associated with a completion rate of only 70% [20]. We did not compare the number of hospitalized patients between both groups. However after excluding patients with an in-hospital CE procedure, which is a known risk factor for incomplete CE procedures [1], the difference between the domperidone and the erythromycin group became even more significant.

A drawback of using erythromycin as a prokinetic agent may be the development of bacterial resistance. However, since erythromycin is given as a single dose, it can be assumed to be associated with a smaller chance of bacterial resistance development compared tot a long course of antibiotics. Similarly, the use of a single dose of erythromycin in the setting of massive upper gastrointestinal bleeding prior to endoscopy is widely accepted [21]. Nevertheless, if an equally effective, non antibiotic, prokinetic agent became available, it would be preferable to use over erythromycin.

Comparing the baseline characteristics in the two groups, we noted that there were slightly significantly more patients with Crohn’s disease in the domperidone group than in the erythromycin group and that the mean age in this group was slightly younger. One could imagine that in patients with strictures or inflammation in Crohn’s disease, the capsule may take more time to pass the small bowel. However, we do not expect that this influenced our results as we showed that the beneficial effect of erythromycin could be attributed to acceleration of the GGT, while SBTT was not significant different between both groups. Looking at the other baseline characteristics both groups were quite similar. Overall this is the largest study to date showing that erythromycin effective increases the rate of complete capsule endoscopy procedures. Potential applications in clinical practice may be to administer erythromycin in patients with expected prolonged GGT or patients with previously incomplete CE investigations. Alternatively, erythromycin may be considered when a real time viewer suggests gastric retention. In our practice, erythromycin is administered prior to every CE. It is likely that technical improvements such as longer battery times will further improve capsule endoscopy completion rates in the future.

## Conclusions

In the largest study to date, orally administered erythromycin prior to CE increased the number of complete capsule endoscopy procedures compared tot domperidone by accelerating the gastric transit time. However, the diagnostic yield was similar in both groups.

## Abbreviations

CE: Capsule endoscopy
GTT: Gastric transit time
SBTT: Small bowel transit time
CI: Confidence interval.
